# Supplementary material for: Physical activity mitigates the link between adverse childhood experiences and depression among U.S. adults
Source: PLoS One. 2022 Oct 12;17(10):e0275185. doi: 10.1371/journal.pone.0275185 (PMC9555628; doi:10.1371/journal.pone.0275185)
Supplement: Attachment — Submitted filename: Response to Reviewers - PONE-D-22-17743.docx [file pone.0275185.s001.docx]

**Response to Reviewers**

***PONE-D-22-17743***

*Editor Comments:*

1. Was pharmacologic treatment (previous or ongoing) a survey variable? Did they undergo or were under therapy? I think you should address that issue under discussion or limitations.

*Response 1:*

Thank you for emphasizing this consideration. The only data for pharmacologic treatment included within the BRFSS dataset was for individuals with a history of cancer or hepatitis B. Please see lines 233-235 of the Discussion section for mention of the lack of a pharmacologic treatment variable as a study limitation.

*Reviewer 1 Comments:*

1. Starting from keywords, you are listed many words and decrease to four to six words, and use only relevant words.

*Response 1:*

Thank you for this feedback. I have now decreased the number of keywords to five key words.

2. In the abstract, you did not write the main objective and significance of the study which is very important to write in the background under the abstract.

*Response 2:*

Thank you for this suggestion. Please see the modifications made to the Abstract, which now includes mention of the study objective and significance.

3. In addition, you took the content of the abstract directly from the body part of your research. But, it should be paraphrased or other similar words.

*Response 3:*

Edits have now been made to the Abstract in an effort to make the abstract unique from similar content in the main manuscript text.

4. Introduction is good. But, in the methodology, the sampling techniques and procedures did not clearly state. so, make it clear. The result, discussion, and conclusion are good. But, state them in a short and precise way.

*Response 4:*

Please refer to lines 73-87 concerning the sampling techniques and procedures. Since the BRFSS is an archival dataset, sampling approaches were restricted to including/excluding participant data from the full BRFSS dataset according to whether the state health agency collected data for ACEs, physical activity, and/or depression. Lines 115-117 detail the listwise deletion approach used to remove missing data, which determined the final sample size for our study.

*Reviewer 2 Comments:*

1. I suggest adding new chapter studies with a representative in vivo sample (as a gold standard) will reinforce the results.

*Response 1:*

Thank you for this suggestion. Lines 208-216 highlight the only known studies (some in vivo, some not) that have previously examined the links between ACEs and depression, ACEs and physical activity, and physical activity and depression. Lines 208-210 specifically discuss the only known study assessing the role of physical activity within the link between ACEs and depression.

2. It does recommend adding an evaluation of the subjects by the clinician to know the reliable or specific depression diagnostic instruments.

*Response 2:*

This is certainly an important consideration. Unfortunately, the BRFSS resource page on CDC.gov does not contain information for the specific depression diagnostic instruments used to inform the depression-related items used for the 2020 BRFSS. Please see lines 226-233 in the discussion detailing the limitations of the one-item instrument used to measure both depression and physical activity.

3. Question: How many people are taking medicine during the survey? Or the exercise? It is a posible confusional variable? Are the authors looking for it?

*Response 3:*

Thank you for bringing attention to this important detail. The only data for participants taking medicine during the survey that is included within the BRFSS dataset is for individuals with a history of cancer or hepatitis B. Please see lines 233-235 of the Discussion section for mention of the lack of a pharmacologic treatment variable as a study limitation.
